# Environmentally-Friendly Extraction of Cellulose Nanofibers from Steam-Explosion Pretreated Sugar Beet Pulp

**DOI:** 10.3390/ma11071160

**Published:** 2018-07-07

**Authors:** Wengang Yang, Yanhong Feng, Hezhi He, Zhitao Yang

**Affiliations:** 1 State Key Laboratory of Pulp and Paper Engineering, South China University of Technology, Guangzhou 510641, China; 201410100308@mail.scut.edu.cn; 2 National Engineering Research Center of Novel Equipment for Polymer Processing, South China University of Technology, Guangzhou 510641, China; pmhzhe@scut.edu.cn; 3 The Key Laboratory of Polymer Processing Engineering of the Ministry of Education, South China University of Technology, Guangzhou 510641, China

**Keywords:** sugar beet pulp, cellulose nanofibers, steam explosion, environmentally-friendly

## Abstract

Cellulose nanofibers (CNFs) with an average diameter of 22 nm were prepared from sugar beet pulp (SBP) via an environmentally-friendly method. Steam-explosion pretreated SBP was treated with hydrogen peroxide (H_2_O_2_) bleaching, high-speed blending, and ultrasonic treatment. Thermogravimetric analysis showed that hemicellulose was partially hydrolyzed in the steam-cooking stage, pectin was removed in the explosion stage, and lignin was removed by H_2_O_2_ bleaching. The removal of non-cellulosic components was confirmed by Fourier-transform infrared (FT-IR) spectroscopy. Morphological analysis showed that steam-explosion pretreatment largely extracted the binder materials of hemicellulose and pectin. This exposed the microfibrillated cellulosic fibers, which promoted subsequent nanofibrillation. X-ray diffraction showed that the CNFs had a crystallinity index of 62.3%. The CNFs had good thermal stability, and thus have potential for use as fillers in polymer matrices. The only chemical reagent used in this green method was H_2_O_2_. Combining H_2_O_2_ bleaching with steam explosion, high-speed blending, and ultrasonic treatment reduced the overall energy consumption and increased the efficiency of the CNFs extraction. The method, therefore, has potential application in industrial processes.

## 1. Introduction

As a common agricultural plant in north-eastern China, sugar beet is usually used to squeeze sugar. The waste from this process is sugar beet pulp (SBP), which is currently mostly applied in animal feed [1]. SBP contains approximately 65–80% polysaccharides, which consist of 40% cellulose, 30% pectin, and 30% hemicellulose based on the dry weight [2]. Many recent studies have focused on obtaining value-added products from SBP. For example, researchers used physical and chemical methods to extract pectin from SBP [3,4]. Another possibility for using SBP is extracting cellulose from its parenchymal cell wall for further use. Cellulose is the most abundant natural polymer and has attracted increasing research attention. Nanocellulose is the cellulosic fiber with at least one dimension less than 100 nm, including cellulose nanofibers (CNFs), cellulose nanocrystals, and bacterial nanocelluloses [5]. CNFs are an important group, possessing characteristics such as renewability, high strength and toughness, low thermal expansion, good biocompatibility, and a larger aspect ratio. These attractive characteristics are additional advantages to other nanoscale materials [6]. CNFs have shown potential applications in many fields, such as nanopapers [7], nanocomposites [8], hydrogels [9] and aerogels [10].

Many studies have investigated the isolation of CNFs. CNFs can be isolated through mechanical processes such as high-pressure homogenization (HPH) that force the suspension through a very narrow channel or orifice using a piston, under a high pressure of 50–2000 MPa [11,12], microfluidization that pumps the cellulose slurry at a constant shear rate through a z-shaped chamber to reach a high shear force [13,14], grinding that passes the cellulose slurry between static and rotating grindstones revolving at approximately 1500 rpm [15,16], and intensive ultrasonication that generates hydrodynamic forces of the ultrasound to defibrillate cellulose fibers [17,18]. Cellulose is generally organized into microfibers and connected with hemicellulose by Van der Waals forces and hydrogen bonds. Lignin is regarded as the matrix in the cell wall. The complexity of this natural structure makes it difficult, inefficient, and energy consuming to fibrillate cellulose into CNFs using only a single mechanical treatment. Therefore, enzyme [19] or alkali-acid [20] pretreatments have been used to remove non-cellulosic materials and facilitate the subsequent mechanical processes. Li et al. (2014) produced CNFs with diameters ranging from 10 to 70 nm from de-pectinated SBP using chemical treatments (alkali treatment and bleaching) and a HPH process [21]. Chemical and Fourier transform infrared (FT-IR) spectroscopy results showed that hemicellulose and lignin were efficiently removed, with the cellulose content correspondingly increasing from approximately 44.96–82.83%. Agoda-Tandjawa et al. (2010) reported adopting acidic and alkaline extraction to remove non-cellulosic polysaccharides of SBP. The resulting samples were treated with mechanical stirring, ultrasonication and HPH to obtain CNFs with a diameter of 2–15 nm and a length of up to 10 µm [22]. Chen et al. (2014) prepared CNFs with widths of 10‒30 nm. A high-speed blender was used to break down the fiber structure of chemical purified cotton fibers, and a subsequent HPH process achieved nano-fibrillation [11]. CNFs with diameters of 10‒25 nm were prepared from two commercial cellulose pulps by combining mechanical fibrillation for the initial refining with a subsequent HPH process [23].

A steam explosion is usually used for extracting fermentable sugars from agricultural waste. Recent studies have also exploited steam explosion for extracting and fibrillating CNFs. Steam explosion involves cooking the pulp in pressurized steam for a certain period of time, and a subsequent rapid release of pressure, resulting in the fiber cell wall being ruptured [6]. Steam explosion also leads to the hydrolysis of hemicellulose to water-soluble monosaccharides and oligosaccharides [24]. Steam explosion has been shown to be an efficient method for extracting cellulose from lignocellulosic materials, and has the ability to isolate CNFs [25,26,27]. However, CNFs obtained by the steam-explosion method are generally non-uniform in size and of poor quality. The rapid release of pressure during steam explosion causes a loose structure of the natural fibers and increases the specific surface area. Therefore, steam explosion facilitates subsequent chemical treatments and improves the removal efficiency of non-cellulosic materials because of the increased reactive surface it causes.

The current study aimed to overcome the defect of non-uniformity in the size of steam-explosion prepared CNFs. To achieve this, a subsequent chlorine-free bleaching agent (H_2_O_2_) was used to remove non-cellulosic components of steam-explosion pretreated SBP, and a combination of mechanical treatments (including high-speed blending and ultrasonic treatment) was used to defibrillate the cellulosic fibers. FT-IR spectroscopy was used to analyze the chemical changes. The changes in morphologies, fiber sizes, crystallinities, and thermal stabilities during the whole process were measured.

## 2. Materials and Methods

### 2.1. Materials and Chemicals

SBP purchased from Linxi Lengsan Sugar Co., Ltd., Linxi, China was pulverized in a sealed crusher (Xulang machinery, Guangzhou, China) at a speed of 25,000 rpm for 1.5 min, and then sieved. The resulting powder sized between 100 mesh and 200 mesh was collected and used for extracting CNFs. H_2_O_2_ (Sinopharm Chemical Reagent Co., Ltd, Beijing, China) was of analytical grade.

### 2.2. Preparation of Cellulose Nanofibers (CNFs)

#### 2.2.1. Steam-Explosion Pretreatment

About 40 g dry weight of SBP powder surrounded by copper mesh was supported by a tripod, to suspend the raw material above the water. This arrangement was then placed in a self-designed steam-explosion reaction apparatus. Approximately 500 mL of water was added to the container to provide steam under high temperature. The SBP was first steam-cooked under optimum conditions at 220 °C for 35 min (the resulting sample was named SBP-SC). During this time, the internal pressure of the steam-explosion reaction apparatus was maintained at 2.4 MPa, and then an electromagnetic valve triggered the steam explosion. The resulting sample was thoroughly washed with water to remove soluble components arising from the degradation, and was then stored in a water-swollen state for further use. Pectin was extracted from the supernatant by adding ethanol, according to Phatak’s method [28]. The steam-explosion pretreated sample was named SBP-S.

#### 2.2.2. Isolation of CNFs

About 20 g dry weight of SBP-S was bleached with 200 mL of 6 wt.% H_2_O_2_ in a beaker. The bleaching procedure was conducted at 80 °C for 24 h at pH 1–2 (adjusted by glacial acetic acid). The bleached sample was thoroughly washed with water until obtaining a filtrate pH of 7, and this sample was named SBP-S-B. This was then diluted to a 0.1 wt.% suspension with distilled water. The suspension was first blended in a high-speed blender at 48,000 rpm for 10 min, and the resulting sample was named SBP-S-B-H. This was then ultrasonically treated in an ice/water bath with a repeated cycle of two seconds working time and three seconds rest for a total of 30 min at an output power of 1000 W, to obtain the CNFs. The name of samples obtained after various stages of treatment is shown in Table 1.

### 2.3. Characterization

#### 2.3.1. Fourier Transform–Infrared (FT-IR) Analysis

FT-IR spectra were recorded using a Fourier-transform infrared spectrometer (VERTEX 70, Bruker, Bremen, Germany). The SBP, SBP-SC, SBP-S, SBP-S-B, and CNFs samples were dried in an oven at 50 °C for 10 h under an air atmosphere, and then ground with potassium bromide in an agate mortar. The resulting powders were pressed into thin pellets which were then dried in an infrared box before testing. FT-IR spectra were collected from these pellet samples in the wavenumber range of 400–4000 cm^−1^.

#### 2.3.2. Thermal Properties Analysis

The thermal stabilities of the samples were determined by thermogravimetric analysis (TGA) using a TG 209 F1 Libra^®^ apparatus (Netzsch, Selb, Germany). All samples were dried in an oven at 60 °C for 12 h prior to analysis. About 5 mg of each sample was put in an alumina crucible without a lid, and then measurements were performed under a nitrogen atmosphere with a gas flow rate of 20 mL min^−1^. The heating range was from room temperature to 600 °C, and the heating rate was 10 °C·min^−1^.

#### 2.3.3. X-ray Diffraction (XRD) Analysis

X-ray diffraction (XRD) patterns were recorded using a Bruker AXS D8 (Bruker, Karlsruhe, Germany) advance X-ray powder diffractometer equipped with Cu *K*α radiation. Segal’s method [29] was used to calculate the crystallinity index of SBP, SBP-SC, SBP-S, SBP-S-B, and the CNFs, according to:

C_Ir_ (%) = (I_002_ ‒ I_am_)/I_002_ × 100 (1)

where C_Ir_ is the relative degree of crystallinity, I_002_ is the peak intensity of the crystalline fraction, and I_am_ is the peak intensity of the amorphous fraction.

#### 2.3.4. Morphology Analysis

Field-emission scanning electron microscopy (FE-SEM) (ZEISS Merlin, Oberkochen, Germany) was used to observe the morphological structures of SBP, SBP-S, SBP-S-B, and SBP-S-B-H, under the condition of vacuum and accelerating voltage of 10 kV. The sample was diluted to 0.01 wt.% with water, which was then ultrasonically treated to achieve good dispersion. A droplet of this dispersion was deposited on a piece of clean mica. The water solvent was allowed to evaporate completely, and the specimen was then coated with gold to prevent charging.

Transmission electron microscopy (TEM-2100F) (JEOL Ltd., Tokyo, Japan) was used to observe the morphology of the CNFs. A droplet of diluted CNFs suspension (0.001 wt.%) was deposited on the surface of a clean copper grid coated with a thin carbon film. The sample was then dried at room temperature before TEM observation. TEM images, which were collected at an accelerating voltage of 200 kV, were used to determine the size of the CNFs with the aid of the Image Pro software package. The diameter distribution of the CNFs was calculated based on the measurement of 500 individual CNFs.

## 3. Results and Discussion

### 3.1. FT-IR Analysis

**Figure 1:**
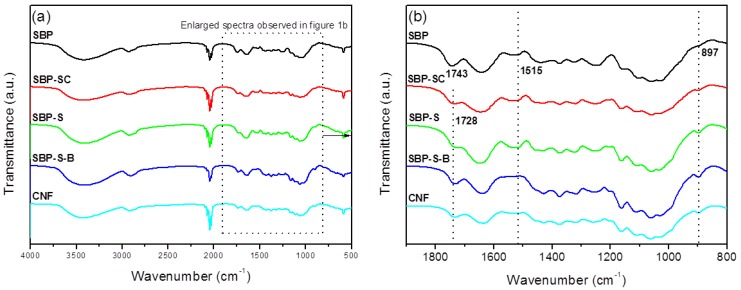
(**a**) Fourier transform–infrared (FT-IR) spectra of sugar beet pulp (SBP) samples SBP, SBP-S, SBP-S-B, and the CNFs; (**b**) enlarged FT-IR spectra between specific wavenumbers.

FT-IR spectra of samples obtained after various stages of treatment are shown in Figure 1. The dominant peaks between 3600 and 2800 cm^−1^ observed in all spectra were due to the stretching vibrations of –OH and –CH groups, respectively [20]. The peak at 1731 cm^−1^ in the spectrum of SBP was due to acetyl and uronic ester groups of hemicelluloses and the ester linkage of carboxylic groups of the ferulic and p-coumaric acids of lignin [24]. This peak was noticeably weaker in the spectrum of SBP-SC, and was also moved to a lower wavenumber (1728 cm^−1^). This was because hemicellulose and pectin were partially hydrolyzed in the steam-cooking stage. The peak at 1728 cm^−1^ was sharper in the spectrum of SBP-S-B. This was due to two effects. On the one hand, H_2_O_2_ oxidized cellulose into an oxycellulose, and therefore some hydroxyl groups of cellulose were substituted with ketone groups [30]. On the other hand, ester groups may also be produced by the reaction of acetic acid and cellulose. Moreover, introducing ester groups with larger steric hindrance to the surface of cellulose also facilitated the subsequent fibrillation. The peak at 1515 cm^−1^ corresponded to the aromatic skeletal vibrations of lignin [31]. This peak became sharper in the spectrum of SBP-S, due to the increase in relative lignin content. The absence of the peak at 1515 cm^−1^ in the spectrum of SBP-S-B indicated that the bleaching process removed the lignin. The peak at 897 cm^−1^ corresponded to the crystalline band of cellulose [32]. This peak became sharper in the spectra as successive treatments were carried out. This was because the relative content of cellulose increased with the removal of non-cellulosic materials. The spectrum of the CNFs was similar to that of SBP-S-B, but the peak at 1728 cm^−1^ was slightly weakened (when using the peak at 897 cm^−1^ as an internal standard for comparing the relative intensity). This indicated the reduction of ester groups to hydroxyl groups.

### 3.2. Thermal Properties

**Figure 2:**
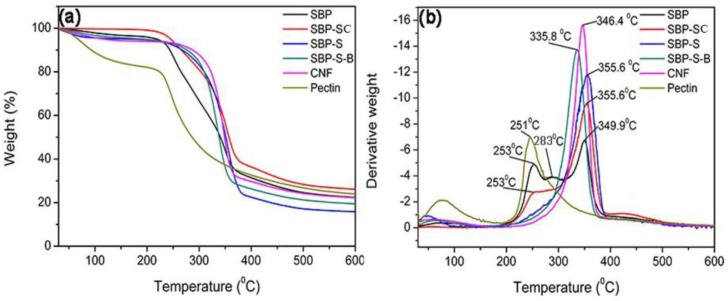
(**a**) Thermal gravimetric (TG) and (**b**) differential thermogravimetric (DTG) curves of SBP, SBP-SC, SBP-S, SBP-S-B, CNFs, and pectin.

Figure 2a,b show thermal gravimetric (TG) and differential thermogravimetric (DTG) curves of samples obtained after various stages in the process of preparing the CNFs. The maximum degradation temperatures of the samples are shown in Table 2. The pyrolysis of hemicellulose and cellulose occurs in the ranges of 220–315 °C and 315–390 °C, respectively [33]. Lignin does not result in obvious pyrolytic peaks because it decomposes over a broader temperature range than the other components [34]. Pectin has a major weight loss in the range of 220–305 °C [35]. By comparing the DTG curves of SBP and pectin extracted from SBP, the peaks at 253 °C, 283 °C and 349.9 °C could be attributed to the pyrolytic peaks of pectin, hemicellulose, and cellulose, respectively. The pyrolytic peak of hemicellulose was absent and the pyrolytic peak of pectin was weak in the DTG curve of SBP-SC. This indicated that most hemicellulose and a fraction of the pectin were hydrolyzed during the steam-cooking. In the DTG curve of SBP-SC, the pyrolytic peak of cellulose shifted to a higher temperature of 355.6 °C. This was due to the removal of hemicellulose, which influenced the thermal stability of cellulose by triggering its degradation at a lower temperature [36,37]. There was still a peak at 253 °C in the DTG curve of SBP-SC, but it was absent in the DTG curve of SBP-S. This indicated that the remaining pectin was mostly extracted in the explosion procedure. The SBP-S-B sample showed a lower thermal stability since the pyrolytic peak of cellulose shifted to a lower temperature of 335.8 °C. This was because the bleaching reagent (H_2_O_2_) lowered the degree of polymerization of cellulose and transformed it into an oxycellulose [30], resulting in the lower thermal stability of cellulose. The thermal stability of the CNFs increased with a higher pyrolytic peak temperature of 346.4 °C, when compared with SBP-S-B. According to FT–IR results, this was due to chemical reduction occurring during high-speed blending and ultrasonication, in which ester groups of cellulose were substituted by hydroxyl groups. Because the bond energy of hydroxyl groups is higher than that of ester groups, more hydroxyl groups would result in more hydrogen bonds and thus more energy would be needed for pyrolysis. As a result, the thermal stability of the CNFs was higher than that of SBP-S-B.

### 3.3. XRD Analysis

**Figure 3:**
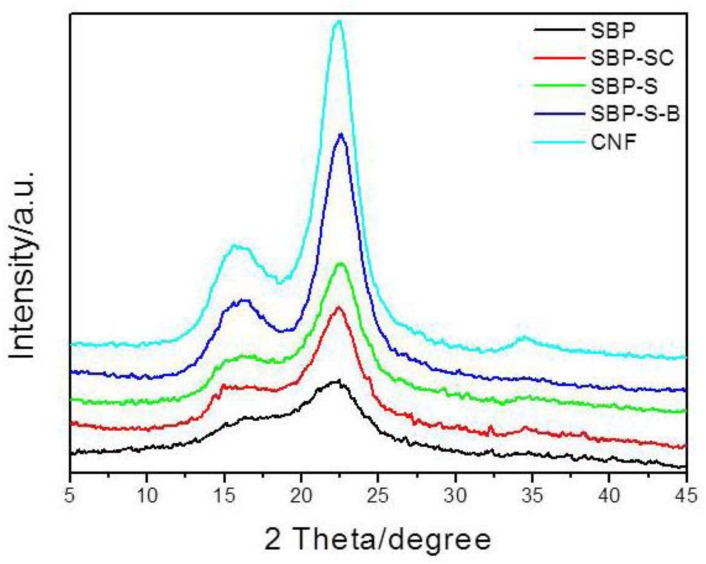
X-ray diffraction (XRD) patterns for SBP, SBP-SC, SBP-S, SBP-S-B, and the CNFs.

XRD patterns of SBP, SBP-SC, SBP-S, SBP-S-B, and the CNFs are shown in Figure 3. All samples exhibited major diffraction intensities at 22.5° and 15.5° 2θ, indicating that all samples were of cellulose I type [38]. The crystallinity indices of the different samples are shown in Table 1. The removal of non-cellulosic components caused the crystallinity index to increase from 29.31 to 59.01%. A small amount of amorphous cellulose was fractioned in the defibrillation process of high-speed blending and ultrasonic treatment, and this part of component was filtered out during samples preparation of XRD analysis. This caused the crystallinity index of the CNFs to increase slightly to 62.30%, compared with 59.01% for SBP-S-B.

### 3.4. Morphological Analysis of the CNFs

**Figure 4:**
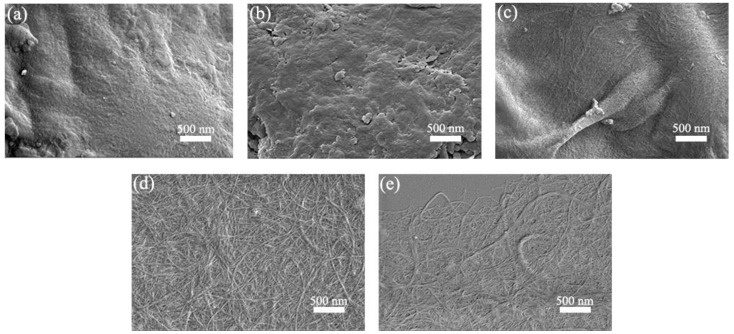
Scanning electron microscope (SEM) images of (**a**) SBP; (**b**) SBP-SC; (**c**) SBP-S; (**d**) SBP-S-B; and (**e**) SBP-S-B-H at 20,000 magnification.

SEM images of SBP, SBP-SC, SBP-S, SBP-S-B, and SBP-S-B-H are shown in Figure 4. Cellulosic fibers are held together by pectin, hemicellulose, and lignin, which are collectively known as the binder materials [39]. Because of this, the cellulosic fibers in the SBP are difficult to discern in Figure 4a. Most hemicellulose and some of the pectin were extracted in the steam-cooking stage. Because of this, the cell wall surface became uneven and the remaining pectin migrated to the surface, as shown in Figure 4b. There were cellulosic microfibers exposed in SBP-S, as can be observed in Figure 4c. This was because the remaining pectin was extracted in the explosion stage, as evidenced by the TGA results. Steam explosion loosens the cell wall structure and partially hydrolyzes hemicellulose and pectin [37]. There were bunches of fibers in SBP-S-B which formed a tight web-like structure, as shown in Figure 4d. This was because lignin in SBP-S-B was removed by the H_2_O_2_ bleaching, which resulted in less binder material. High-speed blending loosened the web-like structure and broke it into smaller pieces, which made the subsequent nanofibrillation process more efficient. Uetani et al. (2010) reported that high-speed blending caused less damage to CNFs compared with grinder treatment [40]. Thus, we used high-speed blending to facilitate the subsequent ultrasonic treatment. Further defibrillation of the cellulosic fibers in SBP-S-B-H can be observed in Figure 4e.

**Figure 5:**
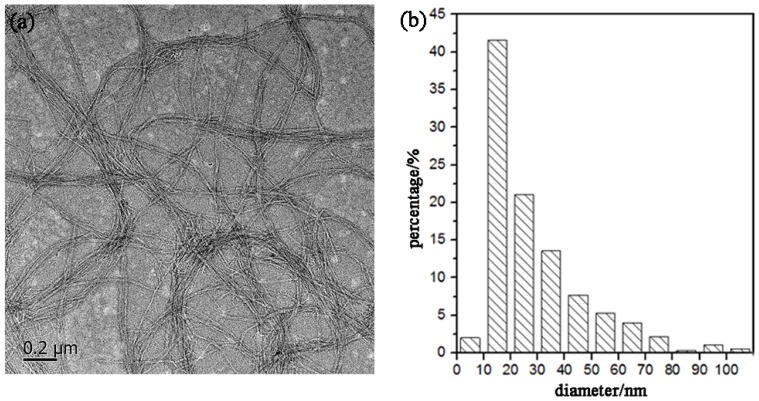
(**a**) Transmission electron microscope (TEM) image and (**b**) diameter distribution of the CNFs.

**Figure 6:**
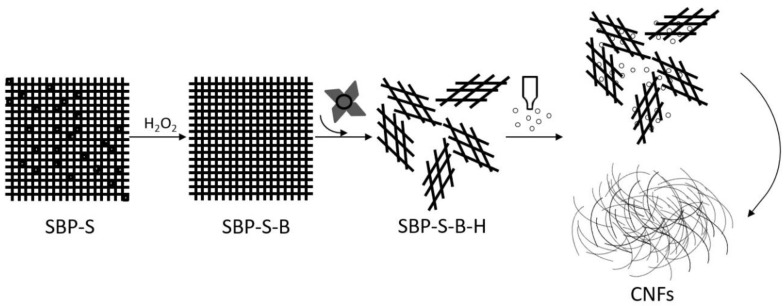
Mechanism of steam explosion, bleaching, high-speed blending and ultrasonic treatment for the extraction of CNFs.

Figure 5 shows a TEM image of the CNFs obtained after ultrasonic treatment, and shows the calculated diameter distribution of the CNFs. The ultrasonic treatment of SBP-S-B-H resulted in defibrillation of the CNFs, and Figure 5a shows the isolated structure of the CNFs. The average diameter was 22 nm. Figure 5b shows that approximately 80% of the CNFs having diameters of 10–50 nm. The mechanism of steam explosion, bleaching, high-speed blending, and ultrasonic treatment for the extraction of CNFs is shown in Figure 6. Steam-explosion pretreatment largely extracted the binder materials and decreased the required use of chemical reagents. Bleaching removed remaining binder materials and exposed the tight web-like structure of the cellulosic fibers. High-speed blending loosened the web-like structure and broke it into smaller pieces. Ultrasonic treatment formed cavitation bubbles to fibrillate these pieces into CNFs. Each treatment played a different but important role in the defibrillation of cellulose, and their combination made the whole process more energy efficient.

## 4. Conclusions

The present study showed that CNFs with an average diameter of 22 nm can be obtained from steam-explosion pretreated SBP, using an environmentally-friendly mechanochemical method involving H_2_O_2_ bleaching, high-speed blending, and ultrasonic treatment. TGA indicated that hemicellulose was mainly hydrolyzed in the steam-cooking stage, and that pectin was mainly hydrolyzed in the explosion stage. FT-IR spectroscopy showed that hemicellulose and pectin in the SBP were removed by steam explosion, and that lignin was removed by H_2_O_2_ bleaching. XRD showed an increase in crystallinity as non-cellulosic components were removed. The maximum degradation temperature of the CNFs was 346.4 °C, which allows the possibility of using these CNFs as fillers in polymer composites. Morphological analysis showed that steam-explosion pretreated SBP had less binder materials than SBP (hemicellulose and pectin), and that microfibrillated cellulosic fibers were exposed during the process. The removal of lignin resulted in the microfibrillated cellulosic fibers forming a tight web-like structure. High-speed blending loosened the web-like structure and broke it into smaller pieces. Ultrasonic treatment formed cavitation bubbles to fibrillate these pieces into CNFs. Most reported studies use bleach pulp as the starting material. In contrast, the current study prepared CNFs from SBP in a process using only one green chemical reagent (H_2_O_2_). The combination of steam explosion, H_2_O_2_ bleaching, high-speed blending, and ultrasonic treatment reduced the overall energy consumption and increased the extraction efficiency. This environmentally-friendly method for preparing CNFs has potential application in industrial processes.

## Figures and Tables

**Table 1:** The name of samples in different stages.

Sample	Treatment
SBP	sugar beet pulp
SBP-SC	steam-cooked sbp
SBP-S	steam-explosion treated SBP
SBP-S-B	bleached SBP-S
SBP-S-B-H	high-speed blending treated SBP-S-B
CNF	cellulose nanofibers

**Table 2:** Maximum degradation temperatures and crystallinity indices of samples at different stages.

Sample	Maximum Degradation Temperature (°C)	Crystallinity Index (%)
SBP	253, 283, 349.9	29.31
SBP-SC	253, 355.6	37.12
SBP-S	355.6	47.79
SBP-S-B	335.8	59.01
CNF	346.4	62.30
